# A Simulation Study on Hypothetical Ebola Virus Transmission in India Using Spatiotemporal Epidemiological Modeler (STEM): A Way towards Precision Public Health

**DOI:** 10.1155/2017/7602301

**Published:** 2017-02-27

**Authors:** Arkaprabha Sau

**Affiliations:** Department of Community Medicine, R. G. Kar Medical College and Hospital, Kolkata, West Bengal, India

## Abstract

*Background*. Precision public health is a state-of-the-art concept in public health research and its application in health care. Application of information technology in field of epidemiology paves the way to its transformation to digital epidemiology. A geospatial epidemiological model was simulated to estimate the spread of Ebola virus disease after a hypothetical outbreak in India.* Methods*. It was a simulation study based on SEIR (Susceptible-Exposed-Infectious-Recovered) compartmental model. Simulation was done in Spatiotemporal Epidemiological Modeler (STEM). Epidemiological profile of Ebola virus, that transmitted throughout the Sierra Leon in 2014–2016, was fitted into the SEIR deterministic compartment model designed for India.* Result*. Spatiotemporal distribution of EVD exposed, infectious, and recovered population at 4-month interval represented by different figures. It is estimated that if no intervention is taken to stop the spread, within 2 years, almost half of the country will be effected by EVD and cumulative number of exposed individuals, infectious persons, and deaths will be 106947760, 30651674, and 18391005, respectively.* Conclusion*. Precision public health may play the key role to achieve the health related targets in the Sustainable Development Goals. Policy makers, public health specialists, and data scientists need to put their hands together to make precision public health a reality.

## 1. Introduction

The incidence of various emerging and reemerging infectious diseases continues to pose a substantial threat to the human health throughout the world [1]. During the past two decades newly emerging ones, for example, severe acute respiratory syndrome (SARS), reemerging ones, for example, West Nile virus, and even deliberately disseminated infectious diseases, for example, anthrax from bioterrorism, threaten the health of the hundreds and millions of the people globally [2]. During early nineties, there was a consensus that it was the time to close the book as the battle against infectious disease had been won [3]. But reemergence of cholera to the Americas in 1991, the plague outbreak in India in 1994, and the emergence of SARS outbreak in 2002-2003, Swine flu (H1N1) pandemic in 2009, and most recently Zika outbreak in Brazil in 2015 eventually prove that thought wrong. Ebola virus disease (EVD) is one of the notorious emerging infectious diseases that endanger the human lives from time to time since its appearance in 1976 in Zaire (later renamed the Democratic Republic of the Congo) and Sudan in Africa continent [2]. The recent epidemic of EVD started in Guinea in December 2013. Within a short period of time, it has spread across land borders to Sierra Leone and Liberia, by air to Nigeria and USA, and by land to Mali and Senegal [4]. On August 8, 2014, the World Health Organization (WHO) declared the EVD outbreak in West Africa a Public Health Emergency of International Concern (PHEIC) under the International Health Regulations (2005). On March 29, 2016, PHEIC related to EVD was lifted from West Africa and on June 9, 2016, WHO declared the end of the most recent outbreak of EVD [5]. By the end of the epidemic, total 15227 confirmed EVD cases have been reported with 11310 deaths in Guinea, Liberia, and Sierra Leone [6]. Till date no indigenous EVD case has been reported in India. But no country is free from the threat of EVD outbreak. A precise prediction about transmission and consequences after an EVD outbreak in India will be effective for proper planning and management to combat with the situation.

Precision public health is a state-of-the-art concept in the new era of public health research and its application in health care. The concept of precision public health evolved within the last two to three years [7]. The precision public health can be simply described as improving the ability to prevent disease, promote health, and reduce health disparities in populations by applying emerging methods and technologies for measuring disease, pathogens, exposures, behaviours, and susceptibility in populations and developing interventional policies for targeted public health programs to improve health [8]. The emergent areas of precession public health are improving methodologies for early detection of pathogens and infectious disease outbreaks, modernizing public health surveillance, epidemiology, and information systems, and targeting health interventions to improve health and prevent diseases [8]. Application of information technology and data science, like real time data acquisition, geospatial epidemiological modelling, big data analytics, and machine learning technology, in field of epidemiology paves the way to its transformation to digital epidemiology, which is conceptually more accurate and precise in nature [8, 9].

Geospatial epidemiological modelling, an application of geographic information system (GIS) [10], is an important tool of precision public health to study the dynamics of disease transmission more accurately. This tool can be applied to predict the spread of an outbreak. Various interventional measures and subsequent outcome can also be studied, which will help to develop efficient and effective disease specific outbreak prevention and management strategies [9].

Keeping the concept of precision public health and geospatial epidemiological modelling in mind, a computer simulation based study, related to hypothetical EVD outbreak in India, was undertaken with following objectives: To simulate the spread of Ebola virus disease after a hypothetical outbreak in India on 01.01.2017 at New Delhi and to predict the number of exposed and infectious persons and deaths due to that EVD outbreak within a span of 2 years.

## 2. Methodology

### 2.1. Simulation Study

Epidemiology is the study of distribution and determinants of health related states or events. One of the most important roles of an epidemiologist is to develop appropriate strategies to prevent or limit the impact of epidemics. Advent of computer based sophisticated technologies helps epidemiologist to study the complex dynamics of disease transmission in silico approach. Mathematical modelling of epidemics can essentially be divided into deterministic and stochastic processes [11]. Deterministic model is based on the average characteristic of the population parameters under study, whereas stochastic model contains randomness of elements. Though stochastic model is more accurate in evaluating real-life epidemic propagation, as it accounts for the randomness of interactions between different population parameters, it is not entirely reproducible. Moreover, when the population is large enough these kinds of randomness neutralise each other and then a simpler deterministic model turns to be good enough to use. Simulation study based on deterministic compartmental model is most commonly applied in epidemiology. This computer simulation is an attempt to model a real-life or hypothetical epidemic situation on a computer so that it can be studied to see how the system works [11]. Infection transmission related different model parameters, commonly applied for compartmental modelling, are shown in Table 1. By changing variables in the simulation, predictions may be made about the behaviour of the system. Incidence of EVD in India is nil, so single reporting of EVD case in India will be considered as an EVD outbreak. A computer based simulation was attempted using Spatiotemporal Epidemiological Modeler (STEM) assuming a single EVD case reported on 01.1.2017 at New Delhi (28.6139°N and 77.2090°E). Then hypothetical spread of EVD in India with time was simulated considering the same dynamics of EVD transmission that was observed in the recent outbreak in West Africa during 2014–2016.

### 2.2. Compartment Model in Dynamics of Disease Transmission

**Figure 1:**
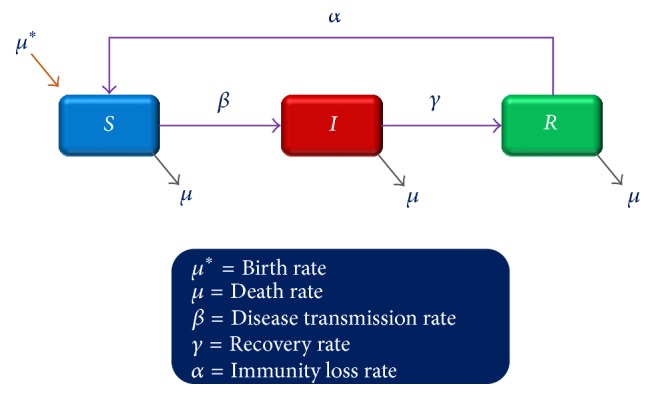
Basic SIR model for disease transmission.

The basic compartment model in epidemiology of disease transmission is SIR model, where “*S*” stands for number of susceptibles, “*I*” stands for number of infectious patients, and “*R*” stands for number of recovered/immune individuals. Disease transmission according to SIR model is depicted in Figure 1. For a fixed population *N*, at a given time *t*, *N* = *S*(*t*) + *I*(*t*) + *R*(*t*). Only those diseases can be explained by this SIR model, which are able to infect others, immediately upon their infection.

**Figure 2:**
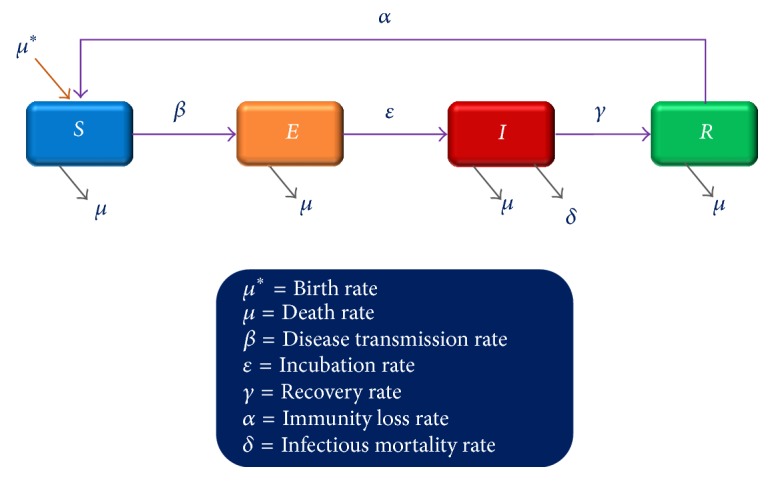
SEIR model for disease transmission.

In case of Ebola virus, humans are not infectious, until they develop symptoms [4]. So to explain the dynamics of Ebola transmission, SEIR model, an extension of SIR model, was adopted, where “*S*” stands for number of susceptibles, “*E*” stands for exposed individuals, “*I*” stands for number of infectious patients, and “*R*” stands for number of recovered/immune individuals (Figure 2).

According to SEIR model at a given time *t*, in *N* number of population, *N* = *S*(*t*) + *E*(*t*) + *I*(*t*) + *R*(*t*). Using ordinary differential equations (ODE), the number of susceptibles, exposed individuals, infectious patients, and recovered/immune individuals at a given time *t* can be predicted from various rate equations

(1) dSdt=−βSNI+αR+μN−SdEdt=βSNI−εE−μEdIdt=εE−γ+μ+ϵ+δIdRdt=γI−αR−μR.

Epidemiological profile of Ebola virus, that transmitted throughout the West Africa in 2014–2016, was fitted into the SEIR deterministic compartment model. Different model parameters described in Table 1 are derived on the basis of disease transmission rate (*β*) 0.128, incubation period was 10 days, infectivity period was 10.38 days, and infectious mortality rate was 70% [6, 12].

There were few assumptions for this model related to this study:

- At the very beginning of the epidemic there was *N*(*t*) = *S*(*t*); that is, total population of India is susceptible as there was no previous incidence of Ebola case.
- Initially, there was a constant number of infectious individuals. *dI*/*dt* = 0. For simulation purpose, it was assumed that one EVD case was reported at New Delhi on 01.01. 2017.
- No specific intervention was initiated to stop the spread of EVD.

### 2.3. Spatiotemporal Epidemiological Modeler (STEM)

It is an open source tool, developed by IBM Corp, available through Eclipse foundation (https://www.eclipse.org/stem/), designed to help public health specialists, data scientists, biomedical researchers, and policy makers to create and simulate spatiotemporal modelling of transmission of emerging and reemerging infectious diseases and distribution of noncommunicable diseases. Public health specialists are responsible for strategies to prevent outbreaks. So they need an accurate understanding of dynamics of disease transmission and the likely outcomes of preventive actions. STEM helps to develop and apply various advanced mathematical models for better understanding of disease dynamics. It comes with a large number of existing compartment models and a new model building framework that allows users to rapidly extend existing models or to create entirely new models. Any STEM model can be run either stochastically or deterministically. Users can choose between many different numerical solvers of ordinary differential equations. When output of a model is fully determined by the initial condition and parameter values, then the model is called deterministic but when same initial conditions and parameter values will lead to an ensemble of different output then the model possesses an inherent randomness that is called stochastic model. Though most of the cases in real world are governed by stochastic processes, this model is more complicated than deterministic model.

In this simulation study, deterministic model with finite difference numerical solver was applied to study the hypothetical spread of EVD in India. Demographic profile of districts according to 2011 Census of India was incorporated into the SEIR model for human to human transmission of EVD.

After developing the model, the programme was run and simulation result in map view was represented at 4-month interval up to 2 years. Then, predicted numbers of exposed individuals, infectious patients, and deaths were logged in data logger.

**Figure 3:**
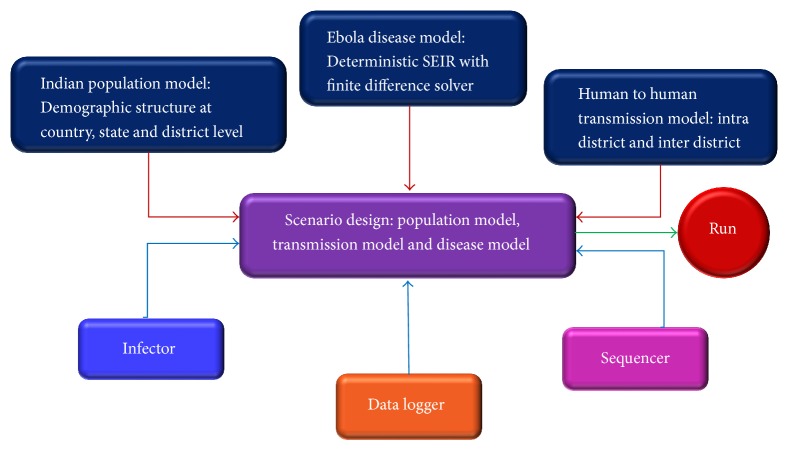
Schema for the simulation study.

Schema of the EVD outbreak simulation with STEM (V 3.0.0 M4) by SEIR model is represented in Figure 3.

## 3. Result

**Figure 4:**
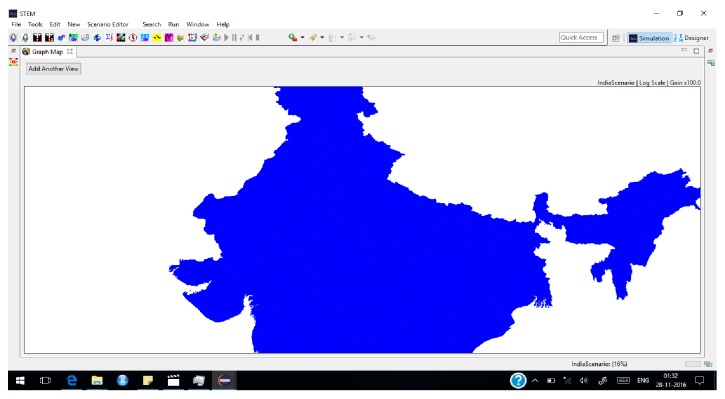
EVD susceptible population as modelled by STEM as on 01.01.2017.

Total population of India is susceptible to EVD as on 01.01.2017 (Figure 4).

Dynamics of hypothetical EVD transmission from 01.01.2017 to 01.01.2019 in India is as follows.

**Figure 5:**
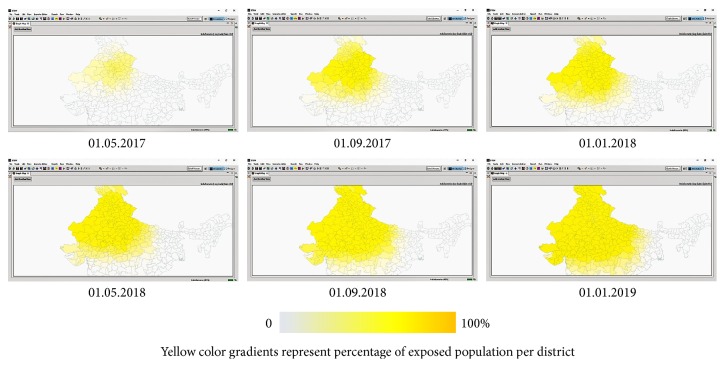
Spatiotemporal distribution of hypothetical EVD exposed population between 01.01.2017 and 01.01.2019.

**Figure 6:**
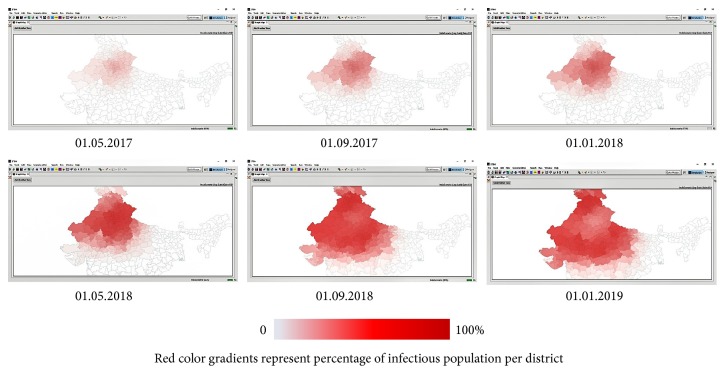
Spatiotemporal distribution of hypothetical EVD infectious population between 01.01.2017 and 01.01.2019.

**Figure 7:**
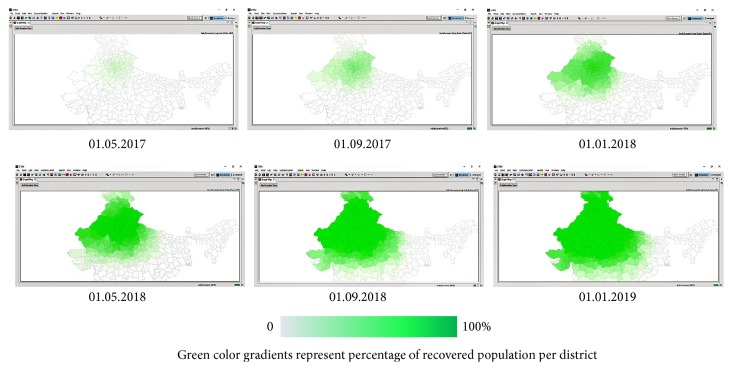
Spatiotemporal distribution of hypothetical EVD recovered population between 01.01.2017 and 01.01.2019.

On 01.01.2017, single case of EVD was reported at New Delhi (28.6139°N and 77.2090°E). Since then, EVD hypothetically spread according to deterministic SEIR model. Spatiotemporal distribution of EVD exposed, infectious, and recovered population at 4-month interval is represented by Figures 5, 6, and 7, respectively.

From STEM data logger, number of effected districts, exposed individuals, infectious persons, and deaths at different time point is shown in Table 2.

## 4. Discussion

Epidemic preparedness is the key factor to minimize the impact of an outbreak. An effective and efficient disease surveillance system can prevent the occurrence of an outbreak. Even if an outbreak occurs, precise and accurate prediction and estimation of its spread and impact is necessary for outbreak management specially in resource allocation and utilization. Another important aspect of epidemic management is generation of public awareness and health education among the susceptible population. Concept of precision public health can be applied in different aspects of epidemic management, that is, preparedness, response, and mitigation.

Recently successful application of precision public health was noticed in the Florida, United States of America (USA), during domestic transmission of Zika virus. Neither the entire USA, nor the entire Florida state, was declared at risk during its notification in July 2016. Instead, precise public health surveillance identified two at-risk areas situated at Miami-Dade County. Mosquito control measures focused on those areas. After six weeks, when the health officials were convinced by ongoing surveillance, restrictions in one area was lifted and resources were mobilized to the other area [9]. So precision public health is a concept that guides precise interventional strategies to address public health problems among precisely defined population using precise surveillance data [13]. Instead of issuing high alert for impending outbreak for entire country at a single point of time, it can be done more precisely at different place at different point of time based on geospatial modelling, simulated using precise surveillance data. Resource mobilization and utilization can be done in a better way. Moreover, unwanted situation like mass panic and its effect on day to day life can be averted.

In this study, concept of precision public health was applied to geospatial modelling of spread and distribution of EVD in India after a hypothetical outbreak at New Delhi on 01.01.2017. From the simulation, it can precisely be estimated about the spread, number of effected districts, infectious population, and probable deaths at different point of time. From this simulation, it is estimated that, within 2 years, almost half of the country will be effected by EVD. It is also possible to model and compare the effect of different interventional strategies at different time frame, to address the epidemic effectively and efficiently. A spatiotemporal modelling of EVD spread in West Africa, done by Pigott et al. in 2016 and Chretien et al. in 2015, assisted the public health specialists in strengthening surveillance and response capacity to contain the EVD outbreak in that area [14, 15]. A computer modelling analysis was done by Merler et al. in 2015 about the spatiotemporal spread of the 2014 outbreak of Ebola virus disease in Liberia and effectiveness of different nonpharmaceutical interventional strategies which helped the Liberian health officials to take necessary actions [16]. In 2016, Burghardt et al. tested and modelled the assumptions related to spatiotemporal spread of recent EVD outbreak in West Africa [17].

In a systematic review on application of spatial methods for infectious disease outbreak, conducted by Smith et al., they found that only 0.4% of the total number of epidemic related published articles between 1979 and 2013 used spatial method for analysis of outbreak spread and identified its scope for much wider implementation [18].

Not only is precision public health confined with the infectious diseases, its application is extended in the field of noncommunicable diseases, genetic diseases, antimicrobial resistance, population screening, and many more [19, 20].

## 5. Conclusion

Geospatial modelling of infectious disease outbreak and precision public health is a new promise in the field of epidemiology. The acquisition and application of precise data and sophisticated geospatial analysis of different epidemiological phenomenon is becoming the routine activity in developed countries. But a large portion of the developing world is not prepared enough to reaping the advantages of precision public health. Precision public health may play a key role to achieve the health related targets in the United Nations' Sustainable Development Goals (SDG). So government leaders, policy makers, biomedical researchers, epidemiologists, public health specialists, global health workers, data scientists, and computer science and information technology experts need to put their hands together to make precision public health a triumph.

## Figures and Tables

**Table 1:** Different parameters in compartmental modelling related to Infection transmission.

Parameters	Description
Susceptible (*S*)	Total number of population who are at risk
Exposed (*E*)	Total number of population who came in contact with a disease person and carrying the infective agent
Infected (*I*)	Number of exposed population developing sign and symptoms and infectious to others
Recovered/immune (*R*)	Number of infected population recovering from the disease and no longer infectious to others. It also includes the population who are resistant to that infection by means of immunization or chemoprophylaxis or previous infection.
Disease transmission rate (*β*)	It is the multiplication of basic reproductive number (*R*_0_) and infectivity rate. *R*_0_ is the number of secondary infections caused by one primary infection introduced to a fully susceptible demographically steady population. Infectivity rate: it is mathematically derived by (1/infectivity period). It is the difference between observed serial number and incubation period.
Incubation rate (*ε*)	It is mathematically derived by (1/incubation period). Incubation period is the time interval between invasion by an infectious agent and appearance of first sign and symptom.
Recovery rate (*γ*)	It is mathematically calculated by (1 − infections mortality rate) × infectivity rate.
Infection mortality rate (*δ*)	It is the percentage of population who died due to that infectious disease.
Immunity loss rate (*α*)	It is derived from the immunity loss period, which is the time interval to became susceptible after complete recovery.

**Table 2:** Number of effected districts, exposed individuals, infectious persons, and deaths.

Date	Total cumulative number			
	Effected district	Exposed	Infectious	Death
01.01.2017	1	1	1	0
01.05.2017	68	16544	3858	2315
01.09.2017	113	107478	29952	14997
01.01.2018	184	547244	167626	100576
01.05.2018	232	3152055	904882	561027
01.09.2018	282	18133878	5185008	3629506
01.01.2019	328	106947760	30651674	18391005
